# Nitrogen Mustard Disrupts Bioenergetics and Activates Oxidative Stress-Induced Cell Death Pathways in Human Keratinocytes

**DOI:** 10.1021/acs.chemrestox.6c00099

**Published:** 2026-05-28

**Authors:** Yi-Hua Jan, Bozena Michniak-Kohn, Laurie B. Joseph, Debra L. Laskin, Jeffrey D. Laskin

**Affiliations:** † Department of Environmental and Occupational Health and Justice, Rutgers University School of Public Health, Piscataway, New Jersey 08854, United States; ‡ Department of Pharmaceutics, 15484 Rutgers University Ernest Mario School of Pharmacy, Piscataway, New Jersey 08854, United States; ∥ Department of Pharmacology and Toxicology, 15484 Rutgers University Ernest Mario School of Pharmacy, Piscataway, New Jersey 08854, United States

## Abstract

Nitrogen mustard (HN2) is a highly reactive bifunctional alkylating agent that causes severe skin injury. To define its impact on keratinocyte bioenergetics and stress responses, we examined mitochondrial function, metabolism, and cell death signaling in human HaCaT cells. Seahorse analysis showed that HN2 caused time- and concentration-dependent suppression of oxidative phosphorylation, including reductions in basal, adenosine 5′-triphosphate-linked, and maximal oxygen consumption. Glycolytic activity was similarly impaired, with decreased extracellular acidification, reduced glucose-stimulated glycolysis, and loss of glycolytic capacity and reserve, indicating broad metabolic dysfunction. HN2-induced bioenergetic impairment triggered rapid nuclear accumulation of nuclear factor erythroid 2-related factor 2 (Nrf2) and upregulation of antioxidant and mitochondrial regulatory genes (HO-1, NQO1, GSTA4, and PGC1α). In parallel, HN2 activated multiple programmed cell death pathways, including apoptosis, autophagy, and ferroptosis, as evidenced by corresponding alterations in Bax, Bcl-xL, LC3-II, SQSTM1/p62, caspase-2, caspase-9, GPx4, TFRC, and ACSL4. Cell cycle analysis identified cells in G2/M as particularly susceptible to HN2, which exhibited enhanced apoptotic signaling. *N*-acetylcysteine attenuated Nrf2 activation, preserved mitochondrial and glycolytic function, and reduced activation of cell death pathways, demonstrating a central role for oxidative and electrophilic stress in HN2 toxicity. These findings reveal a novel mechanism by which HN2 disrupts keratinocyte bioenergetics to drive stress-dependent cell death and highlight antioxidant intervention as a potential strategy to mitigate HN2-induced skin injury.

## Graphical abstract

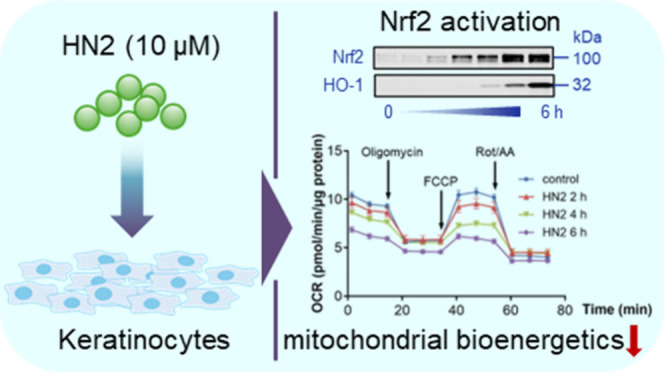

## Introduction

Nitrogen mustard (HN2; mechlorethamine) is a bifunctional alkylating agent structurally related to sulfur mustard (SM), a potent vesicant developed initially for chemical warfare.^1–4^ Both agents react with DNA to form monoadducts, bifunctional adducts, and interstrand cross-links.^5–7^ Beyond nuclear DNA damage, SM accumulates in mitochondria and induces mitochondrial DNA lesions as well as proteins involved in cellular energetics, contributing to mitochondrial dysfunction and oxidative stress.^8–10^ In human bronchial epithelial cells, the SM analogue 2-chloroethyl ethyl sulfide disrupts mitochondrial integrity by reducing membrane potential, triggering permeability transition, and increasing production of reactive oxygen and nitrogen species.^11^ These responses impair oxidative phosphorylation, reduce adenosine 5′-triphosphate (ATP) synthesis, and alter the balance between pro- and antiapoptotic proteins, ultimately leading to activation of cell death pathways.^12,13^ Consistent with these findings, studies in precision-cut mouse lung slices^14^ and primary rat alveolar type II epithelial cells^15^ demonstrate that HN2 markedly suppresses mitochondrial respiration and ATP production and also inhibits glycolytic activity, indicating a broad disruption of cellular energy metabolism.

Keratinocytes are key cellular targets of vesicants,^16,17^ yet the role of mitochondrial dysfunction and metabolic impairment in mustard-induced skin injury remains poorly understood. The skin counteracts stress in a complex manner through integrated antioxidant, metabolic, inflammatory, and repair pathways.^18^ Blister formation is associated with apoptosis and necrosis of basal keratinocytes,^19^ processes that are tightly regulated by mitochondrial integrity and redox homeostasis. Dysfunctional mitochondria generate excess reactive oxygen species, which promote oxidative stress and activate endogenous antioxidant defenses, including those governed by nuclear factor erythroid 2-related factor 2 (Nrf2).^13,20^ Nrf2 controls the expression of cytoprotective genes involved in redox balance, glutathione synthesis, and detoxification of electrophiles and may help mitigate vesicant-induced cellular injury. Although mustard exposure is known to activate Nrf2 signaling,^21^ it remains unclear whether this response can counteract HN2-induced mitochondrial dysfunction and metabolic failure in keratinocytes.

The present studies were therefore designed to delineate the effects of HN2 on mitochondrial bioenergetics, cellular metabolism, oxidative stress, and Nrf2-dependent antioxidant responses in human keratinocytes. By assessing these interconnected pathways, this work provides mechanistic insight into early bioenergetic and redox events driving HN2-induced skin injury and identifies potential targets for therapeutic interventions.

## Materials and Methods

***Caution:*** HN2 is a highly toxic vesicant, and precautions were taken for its handling and preparation including the use of double gloves, safety glasses, masks, and other protective equipment to prevent exposures. Disposal of HN2 waste followed Rutgers University Environmental Health and Safety guidelines.

### Chemicals and Reagents

Dulbecco’s modified Eagle’s medium (DMEM; containing 4500 mg/L d-glucose, 110 mg/mL sodium pyruvate, and 584 mg/L l-glutamine), fetal bovine serum, and penicillin/streptomycin were purchased from ThermoFisher Scientific/Invitrogen (Grand Island, NY). SuperSignal Chemiluminescense Substrates (West Dura, West Pico PLUS), Pierce ECL Western Blotting Substrate, Pierce BCA Protein Assay Kit, Micro BCA Protein Assay Kit, NE-PER Nuclear and Cytoplasmic Extraction Reagents, and Halt Protease and Phosphatase Inhibitor Cocktails were from ThermoFisher Scientific (Rockford, IL). HN2 (mechlorethamine hydrochloride, catalog #122564), oligomycin, antimycin A, rotenone, and all other chemicals were from Sigma-Aldrich (St. Louis, MO) unless otherwise indicated.

### Cells, Treatments, and Protein Preparation

Immortalized adult human keratinocytes (HaCaT) were grown in DMEM supplemented with 10% fetal bovine serum, 100 units/mL penicillin, and 100 μg/mL streptomycin in a humidified atmosphere of 5% CO_2_ at 37 °C. As components in serum, including albumin, are targets for covalent modifications by mustard vesicants, solutions of HN2 were freshly prepared in serum-free DMEM immediately before use. HaCaT cells (5 × 10^6^ cells) were incubated overnight in 15 cm culture dishes and then treated with HN2 (1–50 μM) or vehicle control. These are concentrations of HN2 where early cellular stress responses in keratinocytes can be evaluated including adaptive signaling, mitochondrial dysfunction, oxidative/electrophilic stress, and programmed cell death pathways.^22–24^ After 30 min to 24 h, cells were washed twice with ice-cold PBS (137 mM NaCl, 2.7 mM KCl, 10 mM Na_2_HPO_4_, and 2 mM KH_2_PO_4_, pH 7.4) and removed from the plates with a cell scraper in 5 mL of PBS, and pellets were collected after centrifugation (800*g*, 5 min). Lysates were prepared by sonication of cells on ice with three 10 s pulses in five cell volumes of lysis buffer (PBS containing 0.1% Triton X-100 and 1% phosphatase and protease inhibitor cocktails), followed by centrifugation at 800*g* for 5 min to remove cellular debris. Nuclear fractions were prepared from HaCaT cells using NE-PER Reagents (Thermo Scientific) according to the manufacturer’s protocols. Protein concentrations were quantified with a BCA protein assay kit (Thermo Scientific) with bovine serum albumin as a standard.

### Western Blotting

Protein samples (50 μg) were subjected to reducing and denaturing SDS-PAGE (4–15% Criterion Tris-HCl gel, Bio-Rad, Hercules, CA) followed by electroblotting onto nitrocellulose membranes. Membranes were blocked in 5% nonfat dry milk in PBST (PBS containing 0.1% Tween 20) for 30 min at 37 °C. The blots were incubated with primary antibody (Table 1) overnight at 4 °C followed by HRP-conjugated secondary antibody (Bio-Rad) for 30 min at 37 °C. Specific proteins were visualized using chemiluminescence and the intensity of protein bands quantified using ImageJ software (https://imagej.net/ij/index.html).

**Table 1:** Primary Antibodies Used for Western Blotting

target	host	dilution	source
ACSL4	rabbit	1:10,000	Abcam ab155282
β-actin	mouse	1:25,000	Santa Cruz sc-47778 HRP
Bax	rabbit	1:1000	Cell Signaling #5023
Bcl-xL	rabbit	1:1000	Cell Signaling #2762
beclin-1	rabbit	1:2000	Cell Signaling #3738
caspase-2	rabbit	1:1000	Cell Signaling #2224
caspase-3	rabbit	1:4000	Cell Signaling #14220
caspase-6	rabbit	1:1000	Cell Signaling #9762
caspase-7	rabbit	1:1000	Cell Signaling #9494
caspase-8	rabbit	1:1000	Cell Signaling #4790
caspase-9	rabbit	1:1000	Cell Signaling #9502
caspase-10	rabbit	1:4000	Abcam ab177475
cleaved caspase-6	rabbit	1:1000	Cell Signaling #9761
cleaved caspase-8	rabbit	1:1000	Cell Signaling #9748
cleaved PARP	rabbit	1:4000	Cell Signaling #5625
GPx4	rabbit	1:10,000	Abcam ab125066
GSTA4	rabbit	1:1000	Sigma SAB1401164
HO-1	rabbit	1:10,000	Abcam ab137749
LC3B	rabbit	1:10,000	Cosmo Bio MBL PM036
Nrf2	rabbit	1:1000	Santa Cruz sc-722
Nrf2	rabbit	1:2000	Abcam ab62352
NQO1	rabbit	1:1000	Cell Signaling #3187
PARP	rabbit	1:4000	Cell Signaling #9432
PGC1α	rabbit	1:2000	Cell Signaling #2178
SQSTM1/p62	rabbit	1:4000	Cell Signaling #5114
TFRC	rabbit	1:10,000	Abcam ab84036

### Flow Cytometry

Cells were cultured overnight in 6-well plates (1 × 10^6^ cells/well) followed by treatment with HN2 (10 μM, 50 μM) or vehicle control in a serum-free medium. After 2–6 h, cells were removed from the plates with trypsin-EDTA and washed with PBS containing 1% BSA. Fixation and permeabilization were performed with a FIX & PERM Cell Permeabilization Kit (Invitrogen, Grand Island, NY) according to the manufacturer’s directions. Fixed cells were washed with PBS, incubated in 100 μL of blocking buffer (3% BSA in PBS containing 0.5% Triton X-100) for 15 min at room temperature, and then incubated overnight at 4 °C in the dark with a 1:50 dilution of Alexa Fluor 647 conjugated cleaved caspase-3 (Asp175) antibody (Cell Signaling #9602) or Alexa Fluor 647 conjugated cleaved PARP (Asp214) antibody (Cell Signaling #6987) in blocking buffer. Nuclear DNA was then stained with FxCycle Violet, and cells were analyzed by flow cytometry using a Gallios flow cytometer (Beckman Coulter, Indianapolis, IN). Data was analyzed using the Kaluza software (Beckman Coulter, v2.4).

### Bioenergetics

Oxygen consumption rate (OCR) and extracellular acidification rate (ECAR) were measured using a Seahorse XFe96 Analyzer (Agilent Technologies, Inc., Santa Clara, CA). HaCaT cells, seeded into 96-well Seahorse assay plates (1 × 10^4^ cells/well), were treated with HN2 or vehicle control. After 2–6 h, cells were washed with HBSS, refed with assay medium, and then incubated for 1 h at 37 °C in the absence of CO_2_. OCR and ECAR were recorded under basal conditions before and after sequential additions of metabolic modulators: glucose (25 mM) to stimulate glycolysis; oligomycin (1 μM) to inhibit ATP synthase and assess ATP-linked respiration; carbonyl cyanide 4-(trifluoromethoxy) phenylhydrazone (FCCP, 1 μM) to uncouple mitochondrial respiration and determine maximal respiratory capacity; and rotenone (0.5 μM) plus antimycin A (0.5 μM) to inhibit complexes I and III and quantify nonmitochondrial respiration. In glycolysis stress assays, 2-deoxyglucose (2-DG, 50 mM) was added to inhibit glycolysis and confirm ECAR dependence on glycolytic activity. Bioenergetic parameters were calculated using Agilent Wave software (Agilent Technologies, v 2.6).

### Data Analysis

All data are representative of at least three independent experiments. Data are presented as mean ± SEM and analyzed by the unpaired Student’s *t* test using the GraphPad Prism 10.4.2 software (La Jolla, CA). *P* < 0.05 was considered statistically significant.

## Results

### Effects of HN2 on Mitochondrial Respiration and Glycolytic Function in Keratinocytes

In initial experiments, we analyzed the effects of HN2 on HaCaT cellular bioenergetics by measuring OCR, an indicator of mitochondrial oxidative phosphorylation, and ECAR, a measure of glycolytic activity. HN2 caused a time- and concentration-dependent suppression of basal OCR, consistent with impaired mitochondrial respiratory function (Figure 1A,B). At 10 μM, HN2 reduced basal OCR to 79% of control after 2 h, 61% after 4 h, and 43% after 6 h (Figure 1C), indicating progressive mitochondrial dysfunction. After 4 h of exposure, basal respiration declined to 95%, 61%, and 43% of control at 5, 10, and 20 μM HN2, respectively (Figure 1D), demonstrating dose-dependent inhibition of oxidative phosphorylation. HN2 also diminished glycolytic function in a time- and concentration-dependent manner. Treatment with 10 or 20 μM HN2 attenuated the rapid increase in ECAR normally observed following glucose stimulation (Figure 2A,B), reflecting reduced glycolytic flux. Both glycolytic capacity and glycolytic reserve were significantly decreased across all exposure times (Figure 2C,D), indicating compromised metabolic flexibility and a diminished ability to upregulate glycolysis in response to energetic stress.

**Figure 1:**
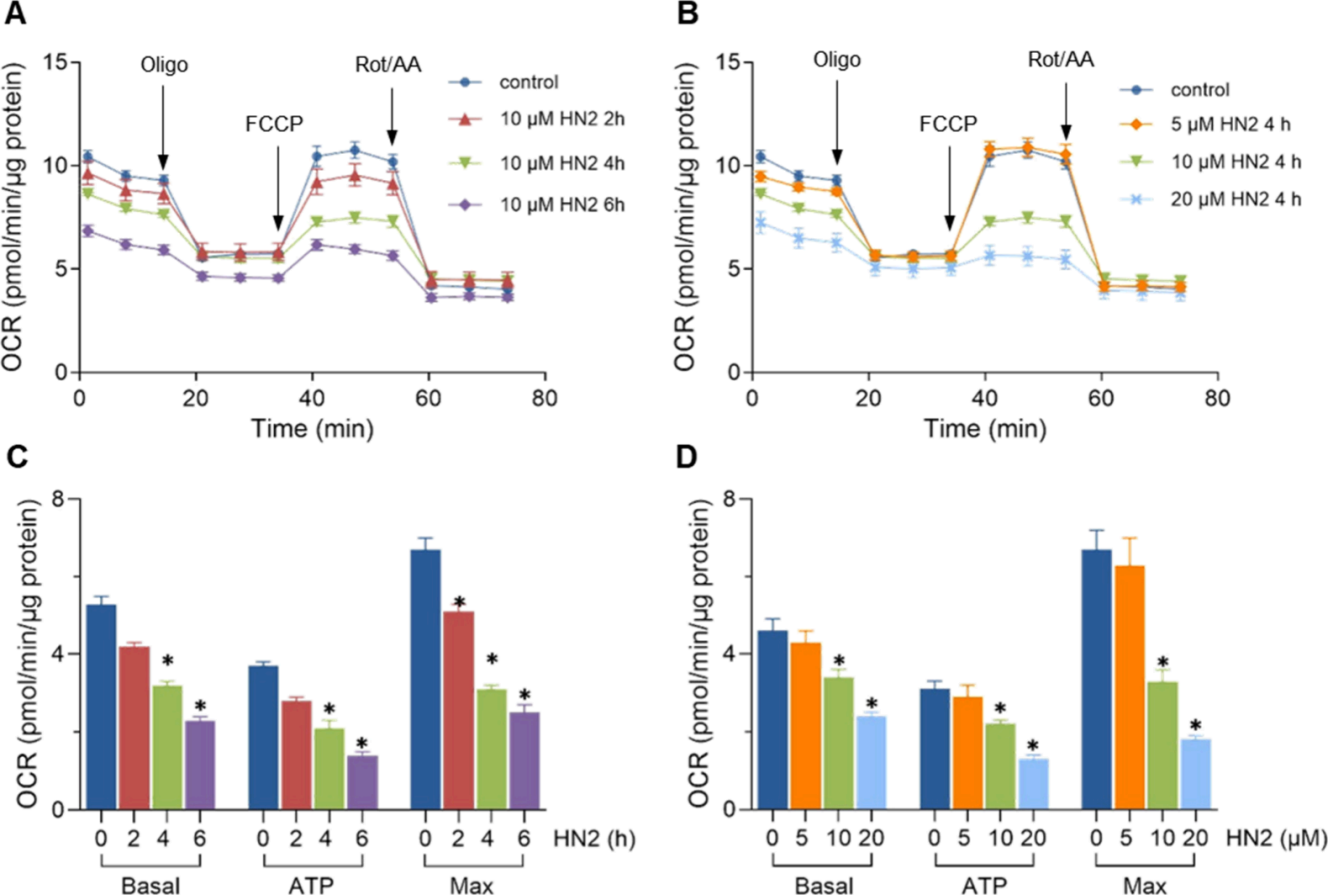
Effects of HN2 on keratinocyte bioenergetics. (A, B) HaCaT cells were treated with HN2 or vehicle control. Oxygen consumption rate (OCR) was analyzed using an Agilent Seahorse XF96 following the sequential addition of oligomycin (Oligo), carbonyl cyanide 4-(trifluoromethoxy) phenylhydrazone (FCCP), and rotenone/actinomycin A (Rot/AA) as described in Materials and Methods. Representative traces from three independent experiments. (C, D) Time-course and dose–response effects of HN2 on basal respiration, ATP-linked respiration, and maximal respiration. Data are presented mean ± SEM (*n* = 6–8). *Significantly different (*p* < 0.05) from vehicle-treated control cells.

**Figure 2:**
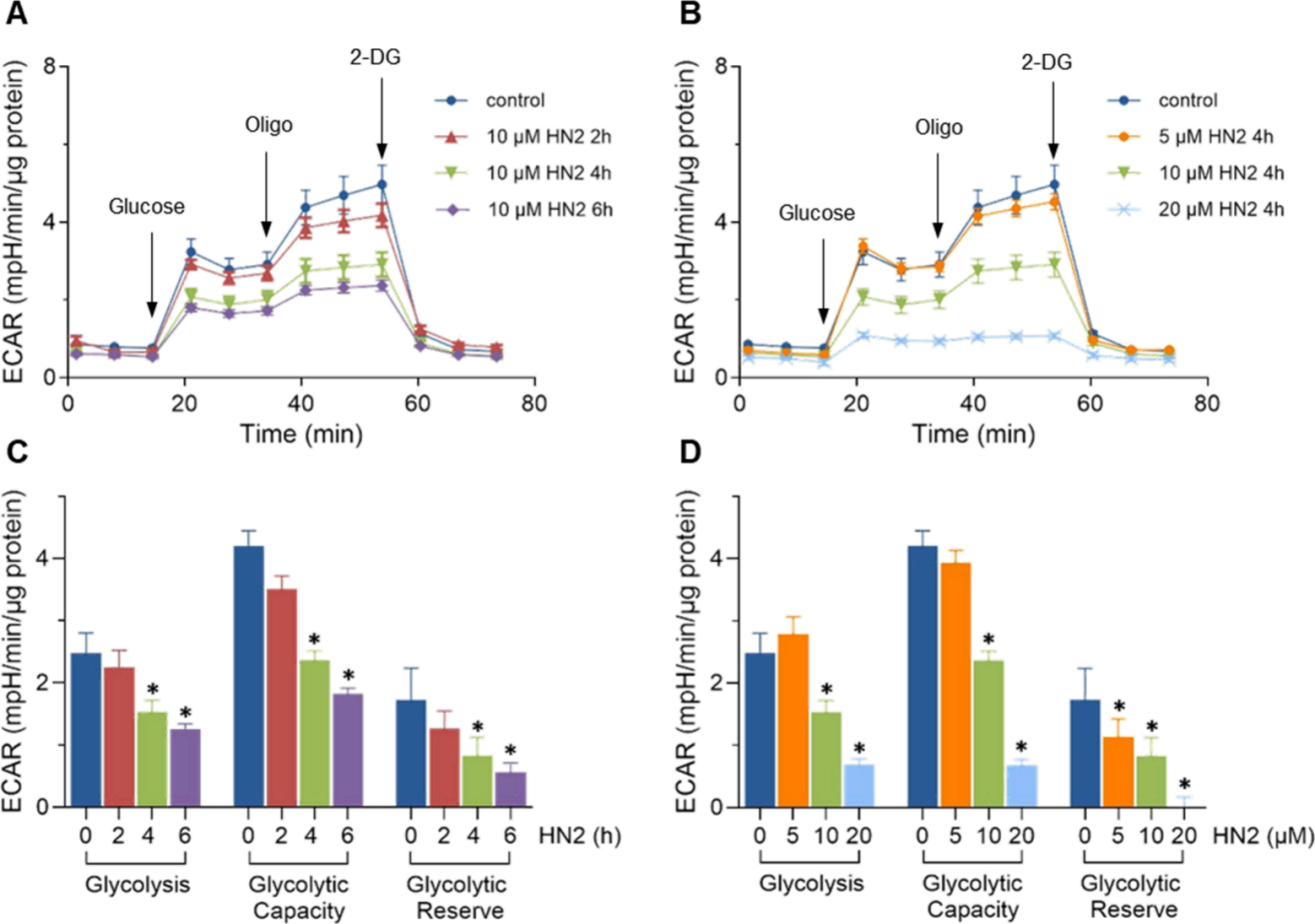
Effects of HN2 on keratinocyte glycolytic activity. HaCaT cells were treated with increasing concentrations of HN2 or vehicle control. (A, B) Extracellular acidification rate (ECAR) was measured using an Agilent Seahorse following the sequential addition of glucose, oligomycin (Oligo), and 2-deoxyglucose (2-DG) at the indicated time points (indicated by arrows). Representative traces from three independent experiments. (C, D) Time-course and dose–response effects of HN2 on glycolysis, glycolytic capacity, and glycolytic reserve. Data are presented as mean ± SEM (*n* = 6–8). *Significantly different (*p* < 0.05) from vehicle-treated control cells.

We next assessed pathway-specific ATP production to determine how HN2 alters cellular energy metabolism. HN2 caused a time- and concentration-dependent reduction in mitochondrial ATP generation, decreasing to 73% of control after 4 h at 10 μM and to 52% at 20 μM (Figure 3A–D). In contrast, glycolytic ATP production remained largely unchanged under these conditions (Figure 3A**–**D), again indicating a limited compensatory glycolytic response. Together, these findings demonstrate that HN2 preferentially disrupts mitochondrial oxidative phosphorylation, leading to impaired energy homeostasis and metabolic stress.

**Figure 3:**
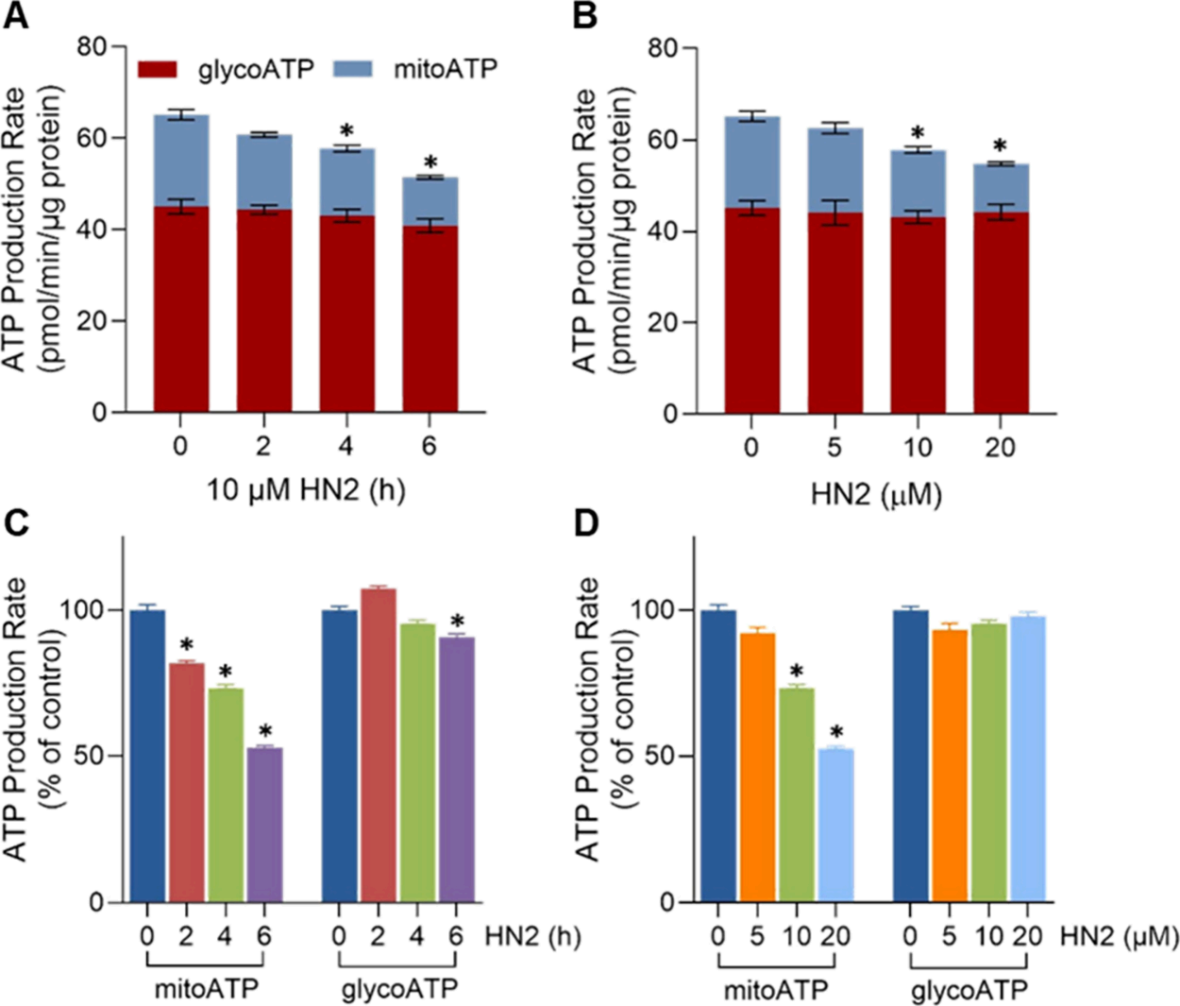
Effects of HN2 on keratinocyte ATP production. HaCaT cells were treated with HN2 or vehicle control. ATP production was measured using the Agilent Seahorse. (A, B) Time-course and dose–response effects of HN2 on ATP production. Glycolysis-mediated ATP production rate (glycoATP) is shown in red, and mitochondrial oxidative phosphorylation-mediated ATP production rate (mitoATP) is shown in blue. Data are presented as mean ± SEM (*n* = 6–8). *Significantly different (*p* < 0.05) from vehicle-treated control cells. (C, D) Effects of HN2 on mitochondrial and glycolytic ATP production.

### HN2 Activates Nrf2 and Induces Antioxidant Gene Expression in Keratinocytes

Because Nrf2 plays a central role in regulating mitochondrial redox homeostasis and cellular stress responses, we next examined the effects of HN2 on Nrf2 signaling. Under basal conditions, Nrf2 is retained in the cytoplasm; upon activation, it translocates to the nucleus to induce target gene expression. HN2 treatment of keratinocytes resulted in a time-dependent accumulation of Nrf2 in the nucleus (Figure 4A), detectable within 1 h following 10 μM HN2, peaking at 6–8 h, and declining thereafter (Figure 4B). Consistent with Nrf2 activation, expression of the canonical Nrf2 target gene heme oxygenase-1 (HO-1) was upregulated in HaCaT cells beginning at 3 h and remaining elevated for up to 12 h before decreasing (Figure 4B). At 24 h, other Nrf2-regulated antioxidant enzymes, including NQO1 and GSTA4, were upregulated (Figure 4C). In parallel, HN2 upregulated expression of the mitochondrial biogenesis regulator PGC1α in a concentration-dependent manner. Concurrent induction of Nrf2 and PGC1α suggests a coordinated adaptive response to HN2-induced mitochondrial and oxidative stress.

**Figure 4:**
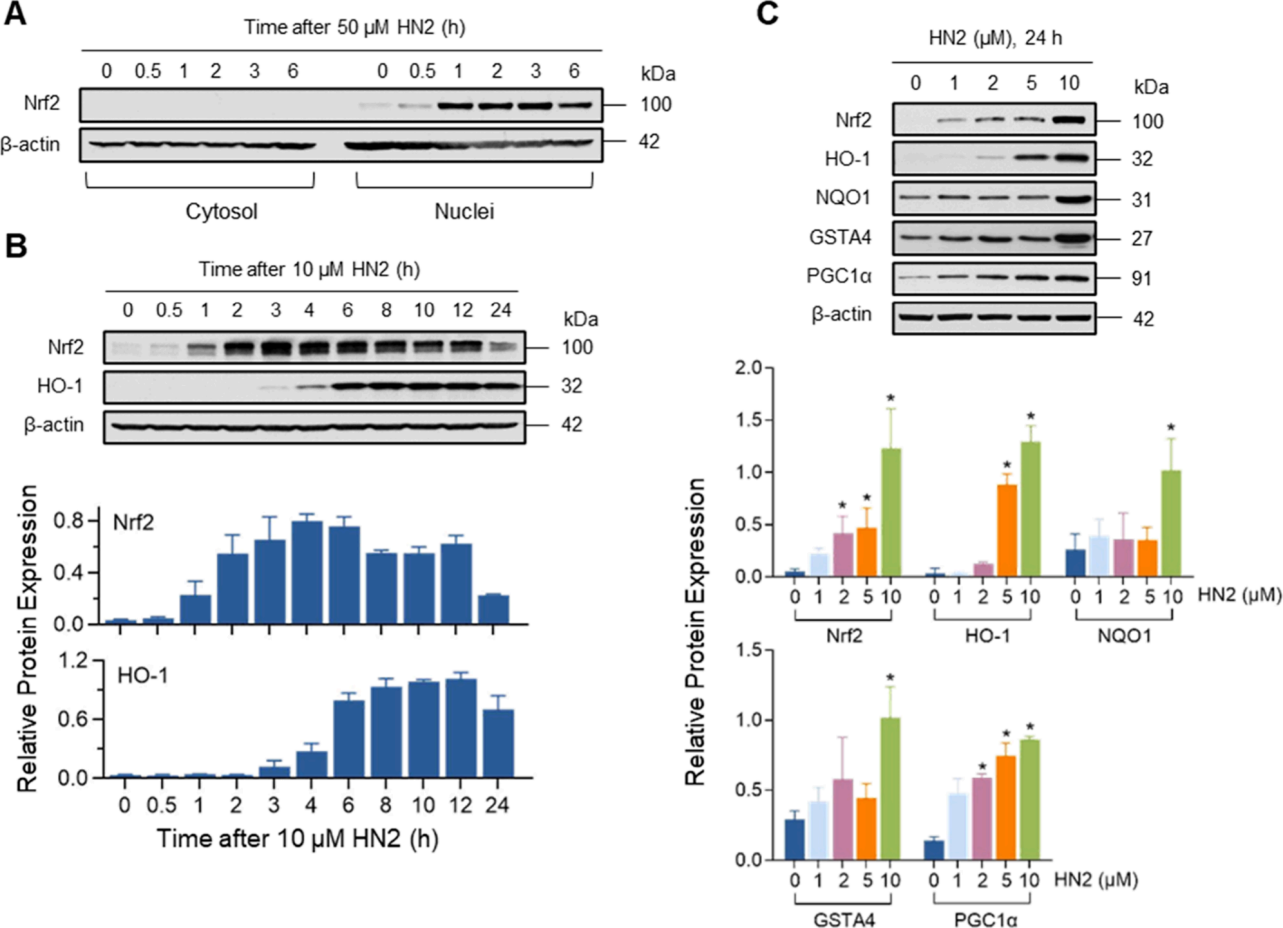
Effects of HN2 on activation of Nrf2 and antioxidant genes. HaCaT cells were treated with HN2 or vehicle control. Lysates were prepared and protein expression analyzed by Western blotting. (A). Effects of HN2 on expression of Nrf2. Cells were treated with 50 μM HN2 for increasing periods of time; cytoplasmic and nuclear fractions were prepared and analyzed for expression of Nrf2. (B, C) Total cell lysates, prepared from cells treated with 10 μM HN2 for increasing periods of time or cells treated with increasing concentrations of HN2 for 24 h, were analyzed for Nrf2 or downstream Nrf2 targets as indicated in the upper panels. Representative Western blots from one of three separate experiments are shown. (B, C, lower panels) Densitometric analysis of Western blots normalized to β-actin and presented as mean ± SE (*n* = 3). *Significantly different (*p* < 0.05) from vehicle-treated control cells.

### Activation of Keratinocyte Death Pathways by HN2

We next examined the effects of HN2 on proteins associated with cell death pathways in keratinocytes. Concentration-dependent increases in expression of the proapoptotic protein Bax and decreases in expression of the prosurvival protein Bcl-xL were observed 24 h after HN2 exposure (Figure 5), indicative of activation of the intrinsic apoptotic pathway. HN2 also upregulated markers of autophagy, including SQSTM1/p62 and LC3-II. Whereas 10 μM HN2 increased SQSTM1/p62 and LC3-II levels by 2.1- and 3.5-fold, respectively, relative to control, the expression of Beclin-1, a key regulator of both autophagy and apoptosis, showed only a modest, nonsignificant increase. HN2 also altered expression of ferroptosis-associated proteins. Specifically, levels of ACSL4 (acyl-CoA synthetase long-chain family member 4) and the transferrin receptor (TFRC) increased, while expression of the antioxidant, GPx4, which catalyzes the reduction of hydrogen peroxide, organic hydroperoxides, and lipid peroxides decreased in a concentration-dependent manner (Figure 5). These data demonstrate that HN2 engages multiple regulated cell death pathways in keratinocytes.

**Figure 5:**
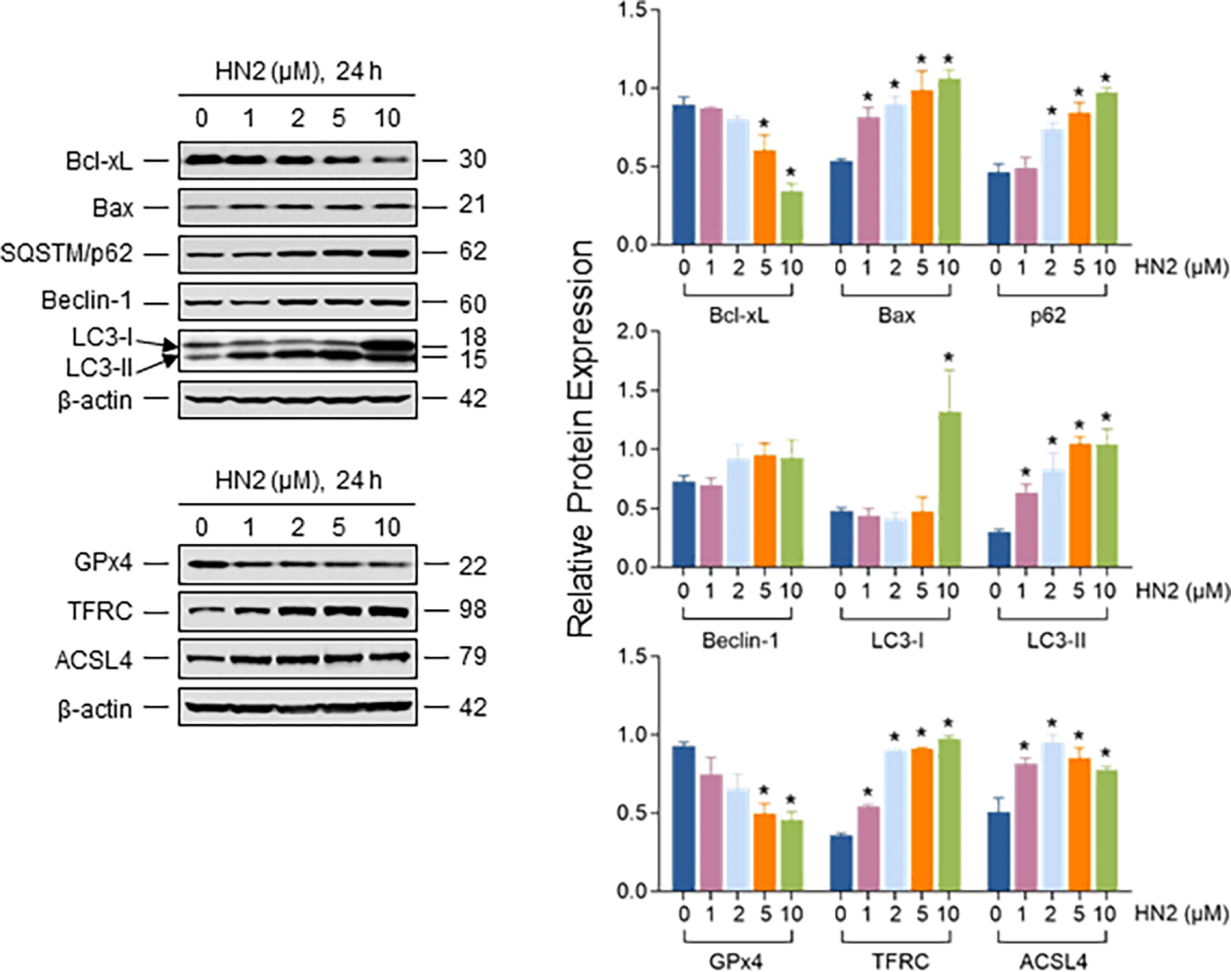
Effects of HN2 on the expression of cell death-associated proteins. HaCaT cells were treated with increasing concentrations of HN2 or vehicle control in serum-free medium. After 24 h, cells were harvested, total cellular lysates were prepared, and protein expression was analyzed by Western blotting. β-Actin was used as a protein loading control. Representative Western blots from one of three experiments are shown. Densitometric analysis of Western blots normalized to β-actin and expressed as the mean ± SE (*n* = 3). *Significantly different (*p* < 0.05) from vehicle-treated control cells.

### HN2 Triggers Apoptosis Predominantly through the Intrinsic Mitochondrial Pathway

To analyze mechanisms mediating apoptosis, we next measured caspase activation, key signaling molecules in the intrinsic mitochondrial pathway. Treatment of HaCaT cells with 10 μM HN2 upregulated cleaved caspase-9, the initiator caspase of the pathway; activation was detectable by 2 h postexposure, peaked at 6–8 h, and declined thereafter (Figure 6A). In contrast, caspase-8 and caspase-10, extrinsic pathway initiator caspases, were not altered at any postexposure time point. Activation of the executioner caspase-3 and cleavage of its downstream substrate, PARP, closely paralleled the kinetics of caspase-9 activation (Figure 6A). At higher HN2 concentrations (50 μM), rapid activation of initiator caspases-2 and -9 and executioner caspases-3, -6, and -7 and PARP cleavage were observed within 3 h of exposure (Figure 6B), whereas caspase-8 and -10 remained unchanged. Similar patterns of caspase activation were observed following 24 h exposure to 1–10 μM HN2. These data demonstrate that HN2-induced apoptosis proceeds primarily through the intrinsic mitochondrial pathway rather than through extrinsic death receptor signaling.

**Figure 6:**
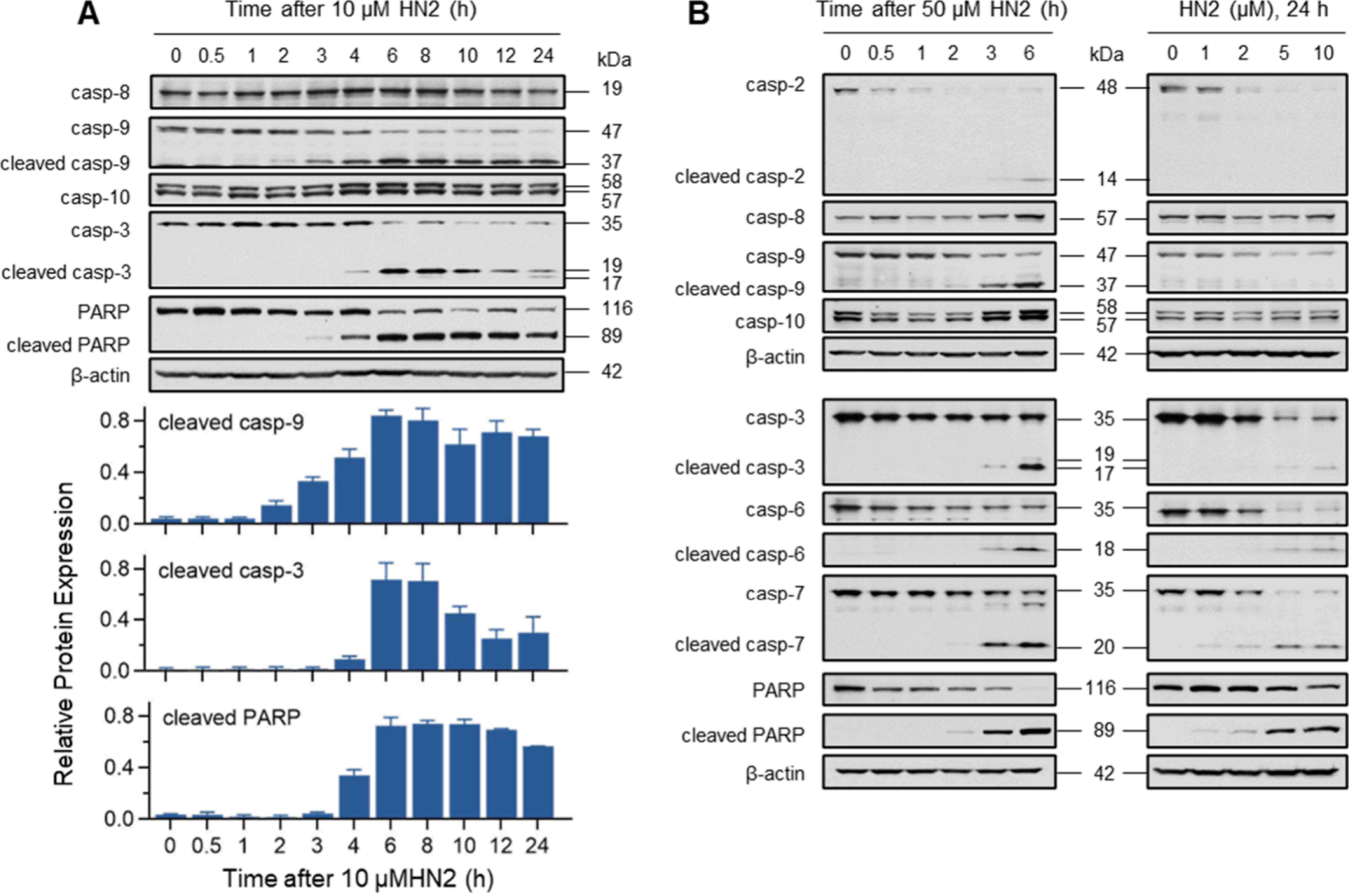
Effects of HN2 on caspase activation. HaCaT cells were treated with HN2 (1–50 μM) or vehicle control. After the indicated times, cells were harvested, total cellular lysates were prepared, and protein expression was analyzed by Western blotting. β-actin was used as a protein loading control. Representative Western blots from one of three separate experiments are shown. (A) Time-dependent activation of caspase 9 and downstream executioner caspases, caspase-3 and PARP, in response to 10 μM HN2. Lower panels show densitometric analysis of Western blots normalized to β-actin and expressed as the mean ± SE (*n* = 3). *Significantly different (*p* < 0.05) from vehicle-treated control cells. (B) Time- and concentration-dependent activation of initiator and executioner caspases following HN2 exposure in keratinocytes.

### HN2-Induced Apoptosis Is Cell Cycle-Dependent and Heightened in G2/M-Phase Cells

Previous studies have demonstrated that alkylating agents, including HN2, induce DNA damage in a cell cycle-dependent manner, with S-phase cells exhibiting greater sensitivity than G1- or G2/M-phase cells.^25^ To determine whether HN2-induced apoptosis is similarly cell cycle-dependent, we analyzed expression of the apoptotic markers cleaved caspase-3 and cleaved PARP across the cell cycle. HN2 treatment resulted in time- and concentration-dependent increases in both markers in all phases of the cell cycle (Figure 7); however, activation was maximal in cells in the G2/M phase. These findings indicate that keratinocytes progressing into mitosis are particularly susceptible to HN2-induced mitochondrial-driven apoptotic signaling.

**Figure 7:**
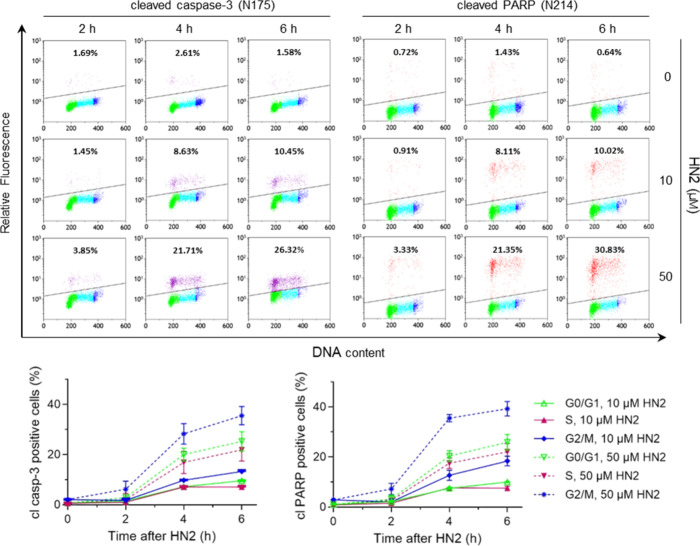
Effects of HN2 on expression of proapoptotic proteins through the cell cycle. HaCaT cells, collected at the indicated time after exposure to HN2 (10 and 50 μM) or vehicle control, were stained with antibodies to cleaved caspase-3 or cleaved PARP followed by FxCycle Violet for DNA content and then analyzed by flow cytometry. Upper panels show representative bivariate distributions of DNA and expression of apoptotic proteins from three independent experiments. Cells expressing cleaved caspase-3 (cl casp-3) are shown in purple, and cells expressing cleaved PARP (cl PARP) are shown in red. Cells in the G0/G1 phase expressing low numbers of immunopositive cells are shown in green, cyan for cells in the S phase, and blue for cells in the G2/M phase. The percentage of immunopositive cells is indicated at the top of each panel. Lower panels show the percentage of immunopositive cells in each phase of the cell cycle. Data are presented as mean ± SE (*n* = 3).

### NAC Protects Keratinocytes from HN2-Induced Bioenergetic Dysfunction and Cell Death

To determine whether oxidative and electrophilic stress contributes to HN2 toxicity, we examined the effects of the antioxidant NAC. Both NAC pre- and post-treatment attenuated HN2-induced Nrf2 nuclear translocation and signaling, as evidenced by reduced expression of the Nrf2 target gene HO-1 (Figure 8A). NAC also suppressed HN2-induced increases in cleaved caspase-3, LC3-II, and TFRC and prevented the loss of GPx4, indicating protection against apoptotic, autophagic, and ferroptotic cell death pathways. Seahorse metabolic analyses showed that NAC alone had no significant effect on mitochondrial respiration, glycolysis, or ATP production (Figure 8B,C,E). In contrast, NAC effectively prevented HN2-induced mitochondrial dysfunction, glycolytic impairment, and ATP depletion, maintaining cellular bioenergetic parameters at levels comparable to untreated controls (Figure 8B–F). These findings indicate that NAC mitigates HN2-induced bioenergetic dysfunction and cell death, at least in part, by limiting oxidative and electrophilic stress and modulating Nrf2 activation. Together, these data highlight a central role for redox imbalance in driving HN2 toxicity and demonstrate that NAC confers substantial metabolic and cytoprotective effects on keratinocytes.

**Figure 8:**
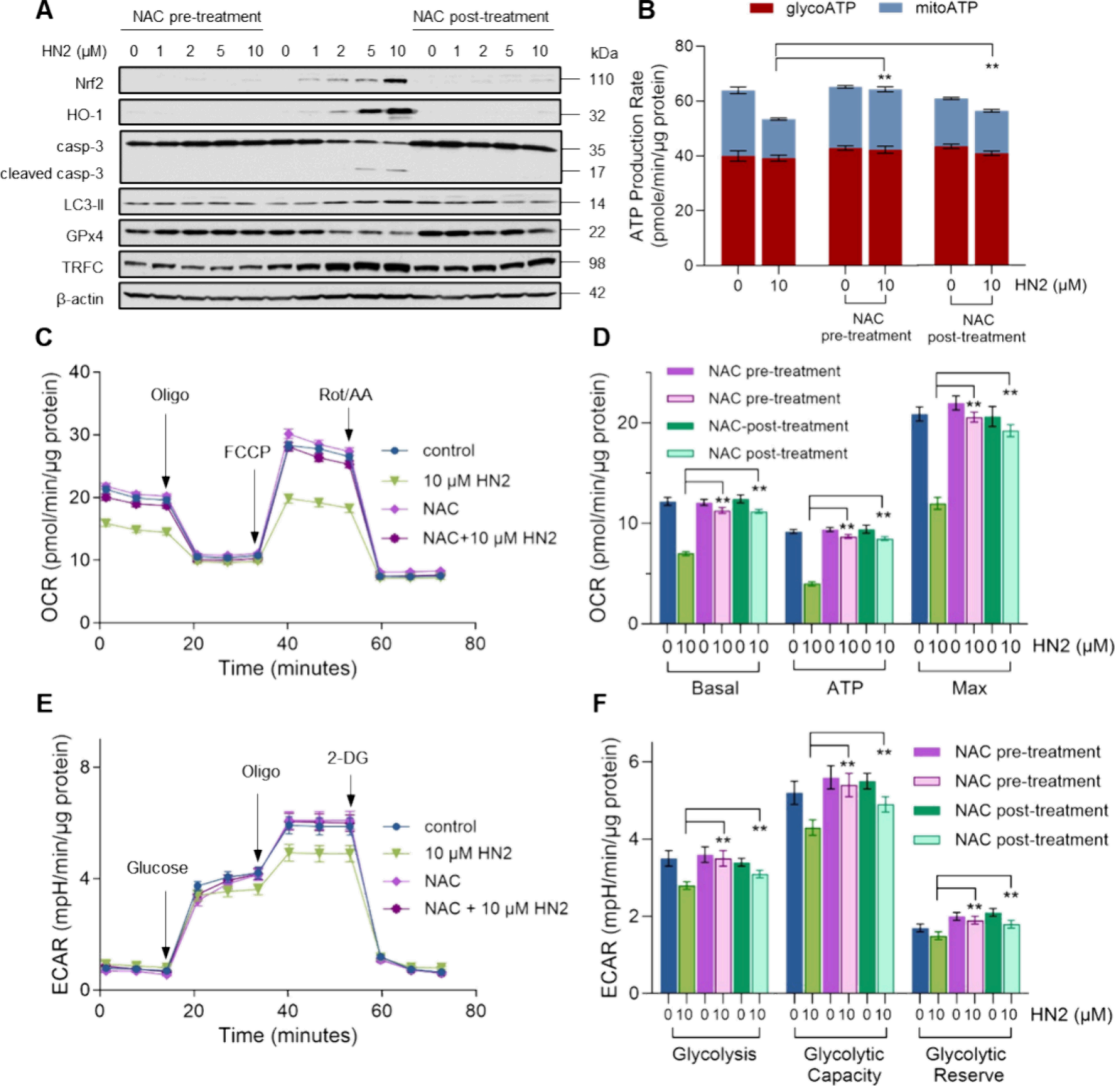
Effects of NAC on HN2-induced alterations in expression of Nrf2, cell death proteins, and metabolism. HaCaT cells were treated with NAC (4 mM) or vehicle in serum-free medium either 30 min prior to or after HN2. Cells were harvested 24 h after HN2, lysates were prepared, and protein expression was analyzed by Western blotting. Seahorse metabolic flux assays were performed 4 h after HN2 treatment, as described in Materials and Methods. (A) Effects of NAC on Nrf2 expression and programmed cell death-associated proteins. Representative Western blots from one of three independent experiments are shown. (B) Effects of NAC on ATP production. Data are presented as mean ± SEM (*n* = 6–8). **Significantly different (*p* < 0.05) compared with cells treated with 10 μM HN2 alone. (C–F) Effects of NAC on HN2-induced mitochondrial and glycolytic stress in keratinocytes. Seahorse traces were from cells pretreated with NAC and compared with cells treated with HN2 alone.

## Discussion

Efficient energy metabolism is essential for keratinocyte survival and for supporting wound healing processes, including cell migration, proliferation, and barrier restoration.^26,27^ Disruptions in cellular bioenergetics exacerbate tissue injury and delay reepithelialization.^26,27^ Studies also suggest that metabolic reprogramming, including shifts in mitochondrial ATP production and glycolytic flux, is a critical component of keratinocyte activation during wound healing.^28,29^ The present studies demonstrate that HN2 causes a marked disruption in the overall energy metabolism in human keratinocytes, a response dominated by reduced mitochondrial oxidative phosphorylation. This resulted in oxidative stress and the activation of multiple cell death pathways. These findings are novel, as they identify a specific molecular target in keratinocytes that is susceptible to HN2-induced metabolic disruption. These data highlight mitochondrial metabolism as a promising target for interventions to reduce vesicant-induced skin injury.

Inhibition of basal mitochondrial respiration in keratinocytes was evident within 2 h of HN2 exposure and progressively decreased over time, consistent with the accumulation of mitochondrial damage. The associated decline in maximal respiration indicates a loss of mitochondrial reserve capacity and an impaired ability to upregulate the oxidative phosphorylation in response to increased energy demands. Because epidermal wound repair depends on intact mitochondrial function,^28^ the loss of respiratory flexibility in keratinocytes following HN2 exposure likely contributes to compromised ATP-dependent processes, such as keratinocyte migration, proliferation, and barrier restoration, thereby impairing wound healing.^29,30^ HN2-induced mitochondrial dysfunction may result from direct alkylation of mitochondrial DNA, electron transport chain proteins, and/or metabolic enzymes. Oxidative stress may also contribute to the pathophysiological response. In this regard, previous studies have shown that HN2 increases the level of reactive oxygen species production and induces structural damage to mitochondria.^11^ These findings suggest that electrophilic injury from HN2 disrupts mitochondrial bioenergetics and undermines the keratinocyte function during wound repair.

In addition to impairing mitochondrial bioenergetics, HN2 disrupted glucose metabolism in keratinocytes. Under conditions that stimulate glycolysis, HN2-treated cells exhibited a blunted ECAR response to glucose with reduced glycolytic capacity and reserve, indicating glycolytic stress and a limited ability to compensate for mitochondrial dysfunction by increasing the glycolytic flux. Despite this, ATP production from glycolysis remained relatively stable, whereas mitochondrial ATP generation declined, reflecting a metabolic shift from oxidative phosphorylation toward aerobic glycolysis.^31,32^ In this state, cells continue to generate ATP rapidly but less efficiently, failing to fully oxidize glucose for maximum energy yield. The limited capacity of glycolysis to compensate for mitochondrial impairment likely contributes to energy depletion, keratinocyte injury, and cell death following HN2 exposure.

Disruption of cellular energy metabolism has been described with other cutaneous toxicants.^33,34^ For example, ultraviolet radiation causes mitochondrial dysfunction, reactive oxygen species generation, and impaired respiration in keratinocytes, paralleling chemically induced bioenergetic deficits.^35,36^ In wound healing models, inhibition of glycolysis and mitochondrial respiration reduces keratinocyte and fibroblast migration and metabolism, highlighting the importance of intact energy metabolism for tissue repair.^37^ Mitochondrial defects also contribute to delayed wound healing in diabetic ulcers, where impaired energy metabolism compromises reparative processes.^38^

Mitochondrial stress is known to activate Nrf2, a key transcriptional regulator of antioxidant and cytoprotective responses.^20,39^ Nrf2-regulated genes contribute not only to antioxidant defense but also to cellular metabolism, DNA repair, and cell survival.^40,41^ Under homeostatic conditions, Nrf2 is sequestered in the cytoplasm by Kelch-like ECH-associated protein 1 (Keap1), which targets Nrf2 for proteasomal degradation.^40,41^ Electrophilic or oxidative stress modifies critical Keap1 cysteines, preventing Nrf2 degradation and allowing its translocation to the nucleus, where it binds to antioxidant response elements (AREs) and initiates a broad cytoprotective program.^41^ Consistent with activation of the Nrf2 pathway, HN2 increased nuclear Nrf2 levels and upregulated canonical Nrf2-dependent antioxidant genes, including HO-1, NQO1, and GSTA4. HN2 also upregulated PGC1α, a key regulator of mitochondrial biogenesis. Although Nrf2 does not directly regulate PGC1α transcription, there is extensive crosstalk between these pathways.^42,43^ Nrf2 activation can indirectly promote PGC1α expression through redox- and energy-sensing mechanisms, while PGC1α reciprocally coactivates Nrf2 to enhance antioxidant and mitochondrial stress responses.^44^ These adaptive responses occur in conjunction with activation of multiple cell death pathways. We found that HN2 upregulated the proapoptotic protein Bax and downregulated the antiapoptotic factor Bcl-xL, increased autophagy markers LC3-II and SQSTM1/p62, and altered ferroptosis-associated proteins,^45^ including ACSL4, TFRC, and GPx4. HN2 also selectively activated the intrinsic apoptotic cascade, as evidenced by cleavage of initiator caspase-2 and -9, without activating extrinsic pathway initiator caspase-8 and -10. Cleavage of PARP, a hallmark of caspase-dependent apoptosis,^46^ was also induced. These results extend prior findings that vesicant-induced DNA damage and oxidative stress activate both intrinsic mitochondrial apoptosis and, under some conditions, extrinsic death receptor pathways.^47,48^ Excessive PARP activation, combined with ATP depletion, may also contribute to necrotic forms of cell death.^49^

Our data also show that HN2-induced apoptosis is cell-cycle-dependent. HaCaT cells in the G2/M phase were more sensitive, as reflected by increased caspase-3 and PARP cleavage, than cells in other phases. This heightened vulnerability likely reflects replication-associated DNA damage and the limited repair capacity at the G2/M checkpoint.^50,51^ Cells entering mitosis with unresolved DNA lesions are prone to mitotic dysfunction, which triggers cell death via intrinsic apoptotic signaling.^52^ In contrast, G1 phase cells more readily undergo cell cycle arrest and DNA repair, rendering them less susceptible to apoptosis. Similar patterns of cell-cycle-specific sensitivity have been reported for other DNA-damaging agents, including platinum-based chemotherapeutics.^53^ Mitochondrial remodeling during the G2/M phase may further increase cellular susceptibility to HN2-induced injury.

To further define the relationship among HN2-induced oxidative stress, Nrf2 activation, and bioenergetic disruption, keratinocytes were treated with the thiol antioxidant NAC.^54^ NAC attenuated HN2-induced Nrf2 activation, as indicated by both reduced nuclear Nrf2 accumulation and decreased expression of the Nrf2 target gene HO-1. NAC also suppressed cleaved caspase-3, LC3-II, and TFRC and prevented the loss of GPx4, indicating protection from apoptotic, autophagic, and ferroptotic cell death pathways. Importantly, NAC prevented the HN2-induced impairment of oxidative phosphorylation, glycolysis, and ATP depletion. These findings indicate that oxidative and electrophilic stress are primary drivers of HN2-induced mitochondrial dysfunction and cell death and that replenishing thiols with NAC disrupts this cascade. Similar cytoprotective effects of NAC have been observed in keratinocytes and other cell types exposed to vesicants and alkylating agents, where thiol loss, mitochondrial damage, and ATP depletion precede the activation of apoptotic or necrotic cell death.^55,56^ Earlier studies in mouse skin have also shown that NAC reduces mustard-induced inflammation, epidermal thickening, basal keratinocyte damage, and microvesication.^57,58^

In summary, these results support a model in which HN2 directly induces electrophilic stress through its reactivity with cellular macromolecules and intracellular antioxidants, leading to depletion of antioxidant defenses and disruption of mitochondrial function and cellular bioenergetics (Figure 9). Both antioxidant depletion and mitochondrial dysfunction promote ROS generation and oxidative stress, which in turn activate adaptive stress-response pathways involving Nrf2 and PGC1α signaling. These pathways stimulate antioxidant defenses, mitochondrial biogenesis, and oxidative phosphorylation in an effort to restore cellular homeostasis. However, when electrophilic and oxidative stress become excessive or sustained, they trigger multiple programmed cell death pathways, including apoptosis, autophagy, and ferroptosis. The ability of NAC to preserve mitochondrial function and attenuate apoptosis, autophagy, and ferroptosis identified oxidative and electrophilic stress as central mediators of mustard toxicity. These results support the use of antioxidant-based interventions as a potential therapeutic strategy to mitigate vesicant-induced skin injury.

**Figure 9:**
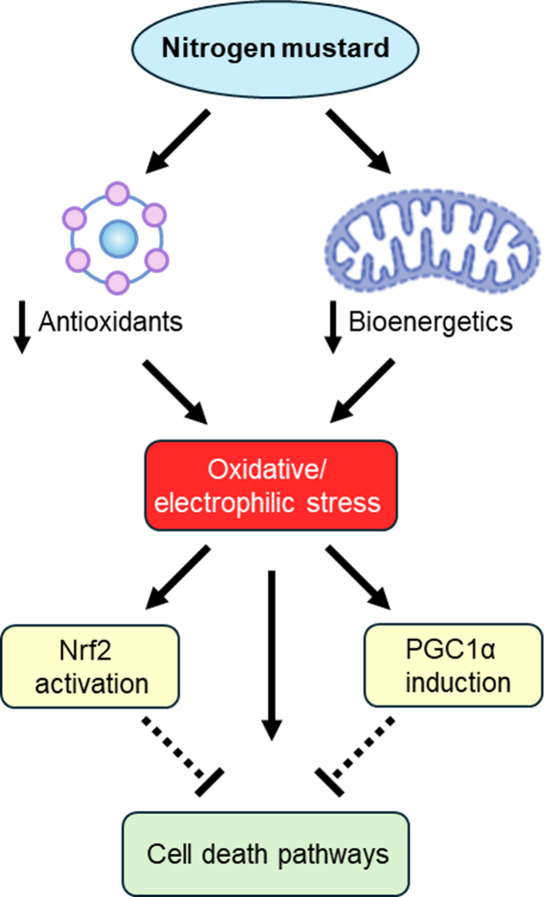
Mechanism of nitrogen mustard HN2-induced oxidative stress and cell death signaling in keratinocytes. NM reacts with intracellular antioxidants, depleting the cell’s defenses against ROS and thereby promoting oxidative stress. NM also disrupts mitochondrial function and impairs cellular energy production, processes that further increase ROS generation. Depletion of antioxidants permits greater NM reactivity within cells and mitochondria, leading to increased electrophilic stress. Together, oxidative and electrophilic stress contribute to mitochondrial dysfunction and activation of cell death pathways. In response to these stresses, cells activate adaptive defense mechanisms, including induction of PGC1α and activation of Nrf2. These pathways promote the expression of genes involved in mitochondrial biogenesis, oxidative phosphorylation, and antioxidant defenses, helping restore cellular homeostasis. Treatment with NAC, a GSH precursor and potent antioxidant, helps preserve intracellular antioxidant capacity and mitochondrial function, thereby protecting cells from oxidative and electrophilic stress-mediated cell death pathways.

## Abbreviations

HN2: nitrogen mustard or mechlorethamine
SM: sulfur mustard
ROS: reactive oxygen species
OCR: oxygen consumption rate
ECAR: extracellular acidification rate
Nrf2: nuclear factor erythroid 2-related factor 2
Keap1: Kelch-like ECH-associated protein 1
NAC: *N*-acetylcysteine
